# Supplementary material for: Asymmetry and changes in the neuromuscular profile of short-track athletes as a result of strength training
Source: PLoS One. 2021 Dec 17;16(12):e0261265. doi: 10.1371/journal.pone.0261265 (PMC8682892; doi:10.1371/journal.pone.0261265)
Supplement: S1 Dataset — (PDF) [file pone.0261265.s001.pdf]

Supporting information file about data of tables 3-6

Muscle	CMJ on both feet [m] before	CMJ on one foot [m] before	CMJ on both feet [m] after	CMJ on one foot [m] after	Power both feet [W] before	Flight time both feet [s] before	CMJ on one foot [m] before	Flight time one foot [s] before	Power both feet [W] after	Flight time both feet [s] after	CMJ on one foot [m] after]	Flight time one foot [s] after
m.BF	0,405	0,23	0,48	0,299	1191,5	0,58	819,9	0,43	1350,1	0,63	966,1	0,49
m.BF	0,337	0,174	0,379	0,235	425,7	0,52	80,5	0,38	514,1	0,56	209	0,44
m.BF	0,285	0,164	0,326	0,217	453,5	0,48	196,5	0,37	540,2	0,52	309,9	0,42
m.BF	0,313	0,203	0,358	0,221	812,3	0,51	578,3	0,41	907,7	0,54	617,3	0,42
m.BF	0,355	0,278	0,372	0,281	1085,2	0,54	921,5	0,48	1120,7	0,55	927,2	0,48
m.BF	0,279	0,186	0,286	0,202	671,1	0,48	472,7	0,39	684,8	0,48	507,9	0,41
m.BF	0,344	0,224	0,365	0,236	509,1	0,53	253,8	0,43	553,8	0,55	281,2	0,44
m.GL	0,405	0,23	0,48	0,299	1191,5	0,58	819,9	0,43	1350,1	0,63	966,1	0,49
m.GL	0,337	0,174	0,379	0,235	425,7	0,52	80,5	0,38	514,1	0,56	209	0,44
m.GL	0,285	0,164	0,326	0,217	453,5	0,48	196,5	0,37	540,2	0,52	309,9	0,42
m.GL	0,313	0,203	0,358	0,221	812,3	0,51	578,3	0,41	907,7	0,54	617,3	0,42
m.GL	0,355	0,278	0,372	0,281	1085,2	0,54	921,5	0,48	1120,7	0,55	927,2	0,48
m.GL	0,279	0,186	0,286	0,202	671,1	0,48	472,7	0,39	684,8	0,48	507,9	0,41
m.GL	0,344	0,224	0,365	0,236	509,1	0,53	253,8	0,43	553,8	0,55	281,2	0,44
m.GM	0,405	0,23	0,48	0,299	1191,5	0,58	819,9	0,43	1350,1	0,63	966,1	0,49
m.GM	0,337	0,174	0,379	0,235	425,7	0,52	80,5	0,38	514,1	0,56	209	0,44
m.GM	0,285	0,164	0,326	0,217	453,5	0,48	196,5	0,37	540,2	0,52	309,9	0,42
m.GM	0,313	0,203	0,358	0,221	812,3	0,51	578,3	0,41	907,7	0,54	617,3	0,42
m.GM	0,355	0,278	0,372	0,281	1085,2	0,54	921,5	0,48	1120,7	0,55	927,2	0,48
m.GM	0,279	0,186	0,286	0,202	671,1	0,48	472,7	0,39	684,8	0,48	507,9	0,41
m.GM	0,344	0,224	0,365	0,236	509,1	0,53	253,8	0,43	553,8	0,55	281,2	0,44
m.GT	0,405	0,23	0,48	0,299	1191,5	0,58	819,9	0,43	1350,1	0,63	966,1	0,49
m.GT	0,337	0,174	0,379	0,235	425,7	0,52	80,5	0,38	514,1	0,56	209	0,44
m.GT	0,285	0,164	0,326	0,217	453,5	0,48	196,5	0,37	540,2	0,52	309,9	0,42
m.GT	0,313	0,203	0,358	0,221	812,3	0,51	578,3	0,41	907,7	0,54	617,3	0,42
m.GT	0,355	0,278	0,372	0,281	1085,2	0,54	921,5	0,48	1120,7	0,55	927,2	0,48
m.GT	0,279	0,186	0,286	0,202	671,1	0,48	472,7	0,39	684,8	0,48	507,9	0,41
m.GT	0,344	0,224	0,365	0,236	509,1	0,53	253,8	0,43	553,8	0,55	281,2	0,44

m.RF	0,405	0,23	0,48	0,299	1191,5	0,58	819,9	0,43	1350,1	0,63	966,1	0,49
m.RF	0,337	0,174	0,379	0,235	425,7	0,52	80,5	0,38	514,1	0,56	209	0,44
m.RF	0,285	0,164	0,326	0,217	453,5	0,48	196,5	0,37	540,2	0,52	309,9	0,42
m.RF	0,313	0,203	0,358	0,221	812,3	0,51	578,3	0,41	907,7	0,54	617,3	0,42
m.RF	0,355	0,278	0,372	0,281	1085,2	0,54	921,5	0,48	1120,7	0,55	927,2	0,48
m.RF	0,279	0,186	0,286	0,202	671,1	0,48	472,7	0,39	684,8	0,48	507,9	0,41
m.RF	0,344	0,224	0,365	0,236	509,1	0,53	253,8	0,43	553,8	0,55	281,2	0,44
m.VL	0,405	0,23	0,48	0,299	1191,5	0,58	819,9	0,43	1350,1	0,63	966,1	0,49
m.VL	0,337	0,174	0,379	0,235	425,7	0,52	80,5	0,38	514,1	0,56	209	0,44
m.VL	0,285	0,164	0,326	0,217	453,5	0,48	196,5	0,37	540,2	0,52	309,9	0,42
m.VL	0,313	0,203	0,358	0,221	812,3	0,51	578,3	0,41	907,7	0,54	617,3	0,42
m.VL	0,355	0,278	0,372	0,281	1085,2	0,54	921,5	0,48	1120,7	0,55	927,2	0,48
m.VL	0,279	0,186	0,286	0,202	671,1	0,48	472,7	0,39	684,8	0,48	507,9	0,41
m.VL	0,344	0,224	0,365	0,236	509,1	0,53	253,8	0,43	553,8	0,55	281,2	0,44
m.VM	0,405	0,23	0,48	0,299	1191,5	0,58	819,9	0,43	1350,1	0,63	966,1	0,49
m.VM	0,337	0,174	0,379	0,235	425,7	0,52	80,5	0,38	514,1	0,56	209	0,44
m.VM	0,285	0,164	0,326	0,217	453,5	0,48	196,5	0,37	540,2	0,52	309,9	0,42
m.VM	0,313	0,203	0,358	0,221	812,3	0,51	578,3	0,41	907,7	0,54	617,3	0,42
m.VM	0,355	0,278	0,372	0,281	1085,2	0,54	921,5	0,48	1120,7	0,55	927,2	0,48
m.VM	0,279	0,186	0,286	0,202	671,1	0,48	472,7	0,39	684,8	0,48	507,9	0,41
m.VM	0,344	0,224	0,365	0,236	509,1	0,53	253,8	0,43	553,8	0,55	281,2	0,44
m.TA	0,405	0,23	0,48	0,299	1191,5	0,58	819,9	0,43	1350,1	0,63	966,1	0,49
m.TA	0,337	0,174	0,379	0,235	425,7	0,52	80,5	0,38	514,1	0,56	209	0,44
m.TA	0,285	0,164	0,326	0,217	453,5	0,48	196,5	0,37	540,2	0,52	309,9	0,42
m.TA	0,313	0,203	0,358	0,221	812,3	0,51	578,3	0,41	907,7	0,54	617,3	0,42
m.TA	0,355	0,278	0,372	0,281	1085,2	0,54	921,5	0,48	1120,7	0,55	927,2	0,48
m.TA	0,279	0,186	0,286	0,202	671,1	0,48	472,7	0,39	684,8	0,48	507,9	0,41
m.TA	0,344	0,224	0,365	0,236	509,1	0,53	253,8	0,43	553,8	0,55	281,2	0,44

Tc [ms] before	Tc [ms] after	Td [ms] before	Td [ms] after	Tr [ms] before	Tr [ms] after	Dm [mm] before	Dm [mm] after	Ts [ms] before	Ts [ms] after
30,37	33,69	27,5	26,18	32,54	43,32	9,75	7,38	186,13	206,39
67,51	50,9	25,37	25,53	56,64	91,13	4,94	6,65	174,5	223,01
17,26	41,97	20,82	25,87	32,49	213,71	1,77	7,46	194,92	331,96
26,51	23,88	22,82	23,34	35,57	28,82	5,14	3,16	189,86	187,97
18,22	22,36	20,66	21,35	49,21	86,06	1,64	2,01	204,27	272,69
23,84	18,34	21,24	22,64	31,52	78,31	1,63	0,77	269,51	335,25
40,09	39,89	25,23	25,34	39,42	36,87	6,58	5,98	164,33	161,53
18,59	21,14	18,29	19,99	43,87	96,51	3,04	3,35	238	298,37
75,89	21,91	22,55	20,25	57,37	38,88	6,35	3,48	170,74	198,31
81,32	27,08	24,22	20,49	33,82	21,87	3,37	3,49	149,92	181,46
28,41	22,99	21,02	19,12	27,74	35,83	6,24	3,95	189,18	227,79
26,04	25,11	19,34	22,62	28,74	19,04	2,31	2,61	189,58	206,02
24,63	25,14	19,99	20,25	47,58	22,86	3,93	2,77	237,22	201,67
58,12	28,1	22,81	21,09	55,39	36,94	10,19	5,91	177,95	195,83
20,36	17,99	19,04	19,9	41,74	58,02	3,17	2,62	231,29	258,66
27,86	24,94	22,38	22,38	92,47	30,55	3,5	3,74	240,97	183,75
23,73	22	20,73	20,76	45,21	46,43	1,89	3,32	188,71	189,76
18,95	21,54	18,94	19,09	74,76	43,7	2,34	3,81	252,71	198,99
27,75	24,17	21,55	22,91	123,03	27,04	3,61	2,65	171,61	187,43
31,64	20,8	21,93	19,71	34,12	33,24	5,19	2,88	157,53	189,17
27,54	24,02	21,19	20,63	31,57	28,43	4,53	2,77	165,35	165,98
45,76	44,63	30,54	34,71	53,93	73,07	17,95	19,06	181,27	213,54
43,62	51,25	32,88	32,29	80,91	66,24	8,4	10,19	249,35	222,78
40,72	61,35	33,94	37,61	103,19	32,03	4	7,52	257,75	280,93
45,27	51,8	34,06	32,68	28,75	44,22	8,29	10,45	184,81	187,51
50,66	54,12	27,6	29,62	39,76	39,84	8,77	8,03	190,4	205,38
48,63	59,77	29,55	30,17	43,18	39,11	8,42	8,5	192,15	183,26
44,36	57,04	31,48	30,82	40,34	47,5	7,9	9,48	170,96	200,63

24,66	23,75	23,37	22,21	134,48	16,8	7,85	6,36	169,68	47,9
29,43	20,94	25,27	23,69	24,45	9,69	6,2	6,11	59,7	31,56
28,38	25,02	23,89	23,56	17,23	15,85	4,72	5,6	56,89	46,97
24,82	26,03	25,04	24,62	17,45	15,39	5,4	5,91	45,23	44,13
38,65	39,3	24,36	24,91	16,57	21,64	6,27	7,03	58,79	65,18
27,29	21,12	24,83	23,52	127,2	14,07	8,64	3,83	161,99	39,46
23,23	22,1	22,84	23,46	84,99	30,29	6,76	5,72	114,46	58,61
25,8	23,26	22,41	20,86	151,42	23,69	8,55	7,4	182,28	51,65
24,02	21,77	22,7	20,04	95,45	44,57	5,13	5,53	134,87	74,86
17,66	17,26	19,89	19,5	13,26	9,87	3,45	3,16	34,37	29,8
20,9	20,48	21,91	20,87	12,29	13,21	6,54	6,69	35,67	36,8
25,17	25,54	22,7	23,86	25,85	30,44	5,95	5,48	54,49	61,77
21,58	21,54	22,08	20,83	15,56	15,44	4,16	3,51	42,06	40,95
23,19	22,65	21,02	20,92	34,6	25,84	7,97	6,05	63,12	54,14
25,78	22,74	21,7	22,33	38,91	228,96	8,13	8,29	231,14	255,39
28,75	22,64	23,79	21,72	72	123,65	6,84	6,95	190,28	184,91
22,58	21,1	24,18	22,68	248,45	146,32	7,95	9,54	389,68	172,34
23,35	22,3	21,28	19,93	38,45	140,42	6,74	6,83	220,81	171,12
25,53	26,63	23,17	23,78	39,36	41,58	8,39	8,65	155,62	182,92
26,02	23,88	23,1	23,04	35,74	45,73	7,9	6,73	186,09	181,94
26,58	23,81	23,19	20,89	24,73	28,05	7,15	6,27	181,54	178,43
19,41	19,11	21,83	21,07	39,79	61,4	4,42	5,52	183,2	249,65
19,03	21,15	21,57	21,29	26,23	40,82	2,26	2,81	214,71	185,03
22,27	21,44	23,9	19,65	35,82	10,54	1,94	2,11	196,39	392,22
19,4	16,94	18,72	18,72	26,69	24,81	2,12	2,11	189,82	237,11
21,55	18,62	21,77	21,97	23,14	24,22	2,67	2,56	191,77	194,97
19,61	17,82	19,51	20,32	26,66	15,47	2,31	2,24	212,62	235,8
21,14	18,7	21,64	20,4	49,85	26,66	2,43	2,67	226,27	203,92
